# EXTREME WEATHER PREPAREDNESS AND CLIMATE ACTIVISM AT AGE 85+

**DOI:** 10.1093/geroni/igad104.3021

**Published:** 2023-12-21

**Authors:** Taylor Patskanick, Sophia Ashebir, Lisa D’Ambrosio, Joseph Coughlin

**Affiliations:** Massachusetts Institute of Technology, Cambridge, Massachusetts, United States; Massachusetts Institute of Technology, Cambridge, Massachusetts, United States; Massachusetts Institute of Technology, Cambridge, Massachusetts, United States; Massachusetts Institute of Technology, Cambridge, Massachusetts, United States

## Abstract

Current projected shifts in climate suggest extreme weather events and disasters will become increasingly common and severe. Older adults are a population vulnerable to the impact of extreme weather due to complex mobility, health, and financial situations in later life, affecting their ability to prepare for and respond to extreme weather emergencies. These compound vulnerabilities demonstrate the importance of adequate emergency preparedness among older adults, particularly among the oldest of older adults or the age 85+ demographic. This paper shares findings from a mixed methods study with the MIT AgeLab 85+ Lifestyle Leaders panel, a research panel of U.S. octogenarians and nonagenarians, on climate change, including their perceptions of generational contributions to climate change, extreme weather preparedness, and engagement in climate justice. Utilizing a survey (n=23) and five virtual focus groups (n=19) conducted in July 2022, findings underscore differences in themes related to weather-event-related emergency preparedness among Lifestyle Leaders living in senior housing versus those community-dwelling. Additional themes note a perceived lack of individual agency and control around impacting climate change locally (including a potential life stage effect) and the urgency of climate change as a societal-level and voting issue. The implications of these findings for effective engagement with the over-85 age demographic in conversations about climate change and emergency preparedness will be highlighted.

